# Short-Term SGLT2 Inhibitor Administration Does Not Alter Systemic Insulin Clearance in Type 2 Diabetes

**DOI:** 10.3390/biomedicines9091154

**Published:** 2021-09-03

**Authors:** Motonori Sato, Yoshifumi Tamura, Hideyoshi Kaga, Nozomu Yamasaki, Mai Kiya, Satoshi Kadowaki, Daisuke Sugimoto, Takashi Funayama, Yuki Someya, Saori Kakehi, Shuko Nojiri, Hiroaki Satoh, Ryuzo Kawamori, Hirotaka Watada

**Affiliations:** 1 Department of Metabolism & Endocrinology, Graduate School of Medicine, Juntendo University, 2-1-1 Hongo, Bunkyo-ku, Tokyo 113-8421, Japan; mo-sato@juntendo.ac.jp (M.S.); hkaga@juntendo.ac.jp (H.K.); n-yamasaki@juntendo.ac.jp (N.Y.); m-kiya@juntendo.ac.jp (M.K.); skadowa@juntendo.ac.jp (S.K.); dsugimo@juntendo.ac.jp (D.S.); tfunaya@juntendo.ac.jp (T.F.); hk-sato@juntendo.ac.jp (H.S.); kawamori@juntendo.ac.jp (R.K.); hwatada@juntendo.ac.jp (H.W.); 2 Sportology Center, Graduate School of Medicine, Juntendo University, 2-1-1 Hongo, Bunkyo-ku, Tokyo 113-8421, Japan; yksomeya@juntendo.ac.jp (Y.S.); skakei@juntendo.ac.jp (S.K.); 3 Clinical Research Center, Graduate School of Medicine, Juntendo University, 2-1-1 Hongo, Bunkyo-ku, Tokyo 113-8421, Japan; s-nojiri@juntendo.ac.jp; 4 Center for Therapeutic Innovations in Diabetes, Graduate School of Medicine, Juntendo University, 2-1-1 Hongo, Bunkyo-ku, Tokyo 113-8421, Japan; 5 Center for Identification of Diabetic Therapeutic Targets, Graduate School of Medicine, Juntendo University, 2-1-1 Hongo, Bunkyo-ku, Tokyo 113-8421, Japan

**Keywords:** SGLT2 inhibitor, insulin clearance, insulin resistance, ectopic fat, hyperinsulinemic euglycemic clamp

## Abstract

Background: Decreased insulin clearance could be a relatively upstream abnormality in obesity, metabolic syndrome, and nonalcoholic fatty liver disease. Previous studies have shown that sodium-glucose cotransporter 2 inhibitor (SGLT2i) increases insulin–C-peptide ratio, a marker of insulin clearance, and improves metabolic parameters. We evaluated the effects of the SGLT2i tofogliflozin on metabolic clearance rate of insulin (MCRI) with a hyperinsulinemic euglycemic clamp study, the gold standard for measuring systemic insulin clearance. Methods: Study participants were 12 Japanese men with type 2 diabetes. We evaluated MCRI and tissue-specific insulin sensitivity with a hyperinsulinemic euglycemic clamp (insulin infusion rate, 40 mU/m^2^·min) before and immediately after a single dose (*n* = 12) and 8 weeks (*n* = 9) of tofogliflozin. We also measured ectopic fat in muscle and liver and the abdominal fat area using ^1^H-magnetic resonance spectroscopy and magnetic resonance imaging, respectively, before and after 8 weeks of tofogliflozin. Results: MCRI did not change after a single dose of tofogliflozin (594.7 ± 67.7 mL/min·m^2^ and 608.3 ± 90.9 mL/min·m^2^, *p* = 0.61) or after 8 weeks (582.5 ± 67.3 mL/min·m^2^ and 602.3 ± 67.0 mL/min·m^2^, *p* = 0.41). The 8-week treatment significantly improved glycated hemoglobin and decreased body weight (1.7%) and the subcutaneous fat area (6.4%), whereas insulin sensitivity and ectopic fat in muscle and liver did not change significantly. Conclusions: MCRI did not change after a single dose or 8 weeks of tofogliflozin. Increased MCRI does not precede a decrease in body fat or improved glycemic control.

## 1. Introduction

Hyperinsulinemia is observed after weight gain and is considered as an important pathophysiological feature of type 2 diabetes mellitus and other metabolic diseases [1]. Indeed, hyperinsulinemia is associated with hypertension, as well as cardiovascular, renal, and liver diseases [1,2,3,4]. While enhanced insulin secretion and reduced insulin clearance could both potentially cause hyperinsulinemia, it has been shown that decreased insulin clearance rather than increased insulin secretion contributes to hyperinsulinemia during weight gain to compensate for insulin resistance [5,6]. Previous reports of genetic deletion and overexpression models of carcinoembryonic antigen-related cell adhesion molecule 1, which promotes receptor-mediated insulin uptake and degradation in hepatocytes, have shown that increased insulin clearance prevents insulin resistance and visceral and liver fat accumulation during over-feeding [7,8]. In addition, experiments of genetic deletion and overexpression models of hepatic insulin-degrading enzyme (IDE) also revealed that impaired insulin clearance due to abnormal IDE directly impairs postprandial hepatic glucose disposal and increases susceptibility to dysmetabolic conditions during the high-fat diet [9,10,11]. Furthermore, previous human studies have demonstrated that low insulin clearance may be the primary mechanism of hyperinsulinemia in black African compared with white European [12,13] and decreased insulin clearance is a risk factor for obesity [14,15], metabolic syndrome [16], and nonalcoholic fatty liver disease (NAFLD) [17,18,19]. Thus, decreased insulin clearance could be a relatively upstream factor that induces metabolic disease and an important therapeutic target for such diseases.

Sodium-glucose cotransporter-2 inhibitors (SGLT2is) are widely used oral hypoglycemic agents in patients with diabetes. These agents are also known to decrease adiposity, NAFLD [20,21,22,23,24,25,26], insulin resistance [24,27], hypertension [28], and adverse cardiovascular [29,30,31,32] and renal outcomes [30,33]. However, the precise underlying mechanisms have not been elucidated. Ferrannini et al. reported that single-dose or 4-week administration of empagliflozin increased insulin clearance [34]. Very recently, similar results were obtained after 24 weeks of tofogliflozin treatment [35]. The mechanisms by which insulin clearance increases after SGLT2i treatment are currently unknown. Accordingly, it has been suggested that increased hepatic insulin clearance is a major regulator of decreased systemic insulin concentrations in the early response to decreased energy and carbohydrate intake [36,37], which is considered as a reasonable biological reaction in terms of glucose homeostasis. Thus, decreased carbohydrate availability by SGLT2i treatment may enhance insulin clearance [36]. Since decreased insulin clearance is considered a relatively upstream inducer of metabolic disease, increased insulin clearance by SGLT2i treatment could be involved, at least in part, in the mechanisms underlying those improvements. However, in previous studies, insulin clearance was calculated as the ratio of total C-peptide concentration to the insulin area under the curve during a meal load test [34,35], a surrogate marker of insulin clearance. Thus, it remains unclear whether SGLT2i treatment alters systemic insulin clearance measured in a hyperinsulinemic euglycemic clamp study, the gold standard for evaluating systemic insulin clearance. Based on these backgrounds, the present study was designed to investigate the effects of single-dose and 8-week SGLT2i administration on systemic insulin clearance as measured by the metabolic clearance rate of insulin (MCRI) in a hyperinsulinemic euglycemic clamp study.

## 2. Materials and Methods

### 2.1. Study Subjects

We screened patients with type 2 diabetes mellitus who regularly attended Juntendo University Hospital in Tokyo, Japan, between November 2018 and May 2019. We selected patients who fulfilled all of the following criteria: (1) male with type 2 diabetes mellitus; (2) glycated hemoglobin (HbA1c) between 6.5% and 10.0%; (3) age between 40 and 65 years; (4) BMI between 18.5 and 30.0 kg/m^2^; (5) stable glycemic control with HbA1c variation <1.0% during the preceding 3 months; and (6) no use of diabetes medications except for α-glucosidase inhibitors and no changes, including dose adjustments, within 12 weeks of consenting to study participation. The following exclusion criteria were applied: (1) type 1 diabetes; (2) heavy alcohol drinking; (3) serious liver disease or hepatitis B or C virus infection; (4) chronic renal failure (estimated glomerular filtration rate <60 mL/min/1.73 m^2^); (5) apparent heart failure or myocardial infarction within the preceding 3 months; (6) history of tofogliflozin use; (7) cancer; and (8) serious diabetic complications, including progressive neuropathy or proliferative retinopathy. All participants gave written, informed consent for study participation. The study was approved by the ethics committee of Juntendo University and carried out in accordance with the principles outlined in the Declaration of Helsinki. The study was registered with the Japan Registry of Clinical Trials (jRCTs031180117, 31 Aug 2019). The study was approved by the ethics committee of Juntendo University and carried out in accordance with the principles outlined in the Declaration of Helsinki.

### 2.2. Study Design

**Figure 1:**
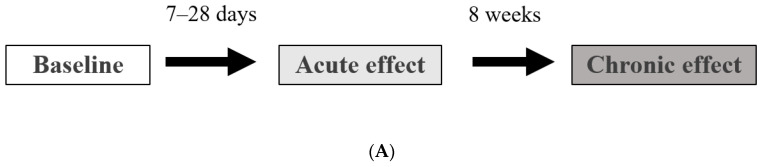

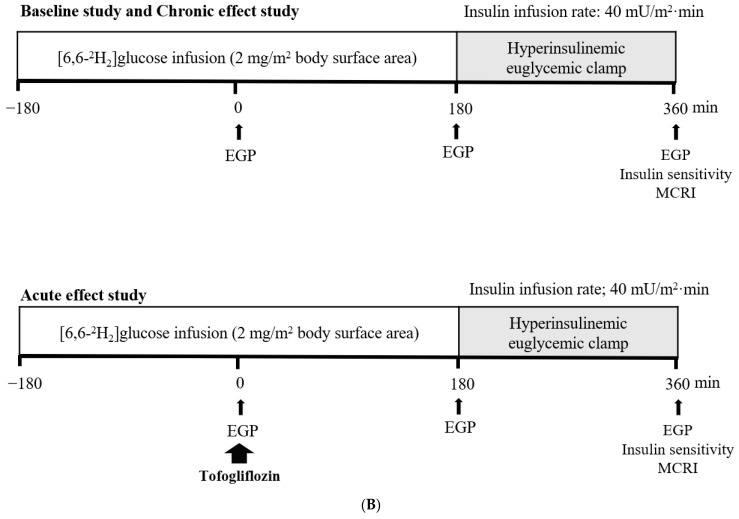
(**A**). Timeline of this study. (**B**) Design of the baseline, acute effect, and chronic effect studies. EGP, endogenous glucose production; MCRI, metabolic clearance rate of insulin.

This study was an open-label, single-arm study to investigate the acute and chronic effects of tofogliflozin on patients with type 2 diabetes mellitus. As shown in Figure 1, all patients visited our institution three times, for the baseline study, acute effect study, and chronic effect study after 8 weeks of tofogliflozin administration. During the baseline study, we infused [6,6-^2^H_2_] glucose for 6 h to measure basal endogenous glucose production (EGP). Next, we performed the hyperinsulinemic euglycemic clamp study for 3 h to quantitate tissue-specific insulin sensitivity and insulin clearance. To assess the effect of a single dose of tofogliflozin on insulin clearance and insulin sensitivity (acute effect study), we infused [6,6-^2^H_2_] glucose for 6 h (−180 to 180 min) at least 7 days after the baseline study. During the [6,6-^2^H_2_] glucose infusion, patients received tofogliflozin (20 mg) 3 h after the beginning of the [6,6-^2^H_2_] glucose infusion. Blood samples were drawn every 30 min for the remaining 3 h (0 to 180 min). Next, we performed the hyperinsulinemic euglycemic clamp study for 3 h (180 to 360 min) to measure tissue-specific insulin sensitivity and insulin clearance. Within 28 days after the baseline study, patients started taking tofogliflozin. After 8 weeks of tofogliflozin treatment, we performed the same clamp study as during the baseline study. During each study, patients urinated at least every 3 h and urinary glucose excretion was measured. In addition, we evaluated body composition using the bioimpedance method, intra-abdominal and subcutaneous fat area using magnetic resonance imaging (MRI) (Siemens, Erlangen Germany), and intramyocellular lipid (IMCL) and intrahepatic lipid (IHL) using ^1^H-MR spectroscopy (^1^H-MRS) at baseline and after 8 weeks of tofogliflozin treatment.

To assess the effect of tofogliflozin on EGP at 180 min after a single-dose administration of tofogliflozin, EGP and other related parameters during −180 to 180 min of the baseline and acute effect studies were evaluated; results have been reported elsewhere [38]. Some of the previous data are presented again in the present study to show the participants’ metabolic status before the hyperinsulinemic euglycemic clamp study.

Primary outcomes of the present study were effects of a single administration of tofogliflozin and 8 weeks of tofogliflozin treatment on MCRI evaluated with a hyperinsulinemic euglycemic clamp study, respectively. Secondary outcomes were changes in body weight, body composition, visceral and subcutaneous fat areas, ectopic fat in muscle and liver, EGP, and tissue-specific insulin sensitivity after 8 weeks of tofogliflozin treatment. The present study was the first study that evaluated the MCRI before and after SGLT2i treatment by the clamp study; thus, we could not perform a power analysis. In the present study, we hypothesized that increased insulin clearance by SGLT2i treatment could have occurred prior to metabolic changes such as body weight reduction and improved glycemic control. Thus, we referred to a similar previous study that used the hyperinsulinemic euglycemic clamp and showed significant metabolic changes after SGLT2i (dapagliflozin) treatment [39], and set the same number of patients (*n* = 12) in the present study.

Two patients withdrew consent after the acute effect study and one patient was excluded due to insufficient treatment. This patient stopped taking tofogliflozin after 4 weeks of treatment. Nine patients completed the chronic effect study. None of their concomitant medications had changed from at least 3 months prior to the baseline evaluation to the end of the study.

### 2.3. Measurement of EGP, Insulin Clearance, and Tissue-Specific Insulin Sensitivity

All measurements were performed at Juntendo University Hospital (Tokyo, Japan) from December 2018 to August 2019. As shown in Figure 1, patients were instructed to consume a standard diet for the 3 days immediately preceding each clamp study. In addition, they were asked to refrain from alcohol 1 day before the clamp studies. After an overnight fast, a hyperinsulinemic euglycemic clamp study was performed with an artificial endocrine pancreas (STG 55; Nikkiso, Shizuoka, Japan). Briefly, after securing an intravenous cannula in the forearm, a bolus dose of [6,6-^2^H_2_] glucose (200 mg/m^2^ body surface area (BSA)) (Cambridge Isotope Laboratories, Tewksbury, MA) was injected intravenously, followed by constant infusion of 2 mg/m2 BSA per minute for 6 h (−180 to 180 min) to measure EGP [40]. After 6 h of [6,6-^2^H_2_] glucose infusion, we infused primed insulin (160 mU/m^2^ per minute for 5 min followed by 80 mU/m^2^ per minute for 5 min) and resumed continuous insulin infusion at 40 mU/m^2^ per minute for 3 h (180 to 360 min). Levels of [6,6-^2^H_2_] glucose infused were decreased to 85% of the basal rate during the hyperinsulinemic euglycemic clamp study to maintain constant plasma glucose enrichment [41]. We used a warming blanket for arterialization of the hand vein. Plasma glucose levels in arterialized blood were maintained at approximately 95 mg/dL with variable infusion rates of 20% glucose containing approximately 2.5% [6,6-^2^H_2_] glucose monitored using an Antsense III glucose analyzer (Horiba, Ltd., Kyoto, Japan). Blood samples were drawn for biochemical analysis at 10-min intervals at 30 min before the clamp study and during the steady-state periods of the clamp study. Enrichment of [6,6-^2^H_2_] glucose in plasma was measured using high-performance liquid chromatography (LTQ-XL-Orbitrap mass spectrometer; Thermo Fisher Scientific, Waltham, Massachusetts), as described previously [42]. A steady-state equation was used to calculate the rate of EGP and rate of the disappearance of glucose (Rd) [43]. The rate of tissue glucose uptake (TGU) was calculated by subtracting the rate of urinary glucose excretion during the glucose clamp study from Rd. We evaluated EGP at 0, 180, and 360 min. We calculated %reduction of EGP (EGP at 0 min—EGP at 360 min/EGP at 0 min) during the hyperinsulinemic euglycemic clamp study divided by steady-state serum insulin (SS_SI_) and used it as an index of hepatic insulin sensitivity [17]. Similarly, TGU was divided by SS_SI_ and multiplied by 1000 and used as an index of muscle insulin sensitivity [44]. Adipose tissue insulin sensitivity was evaluated by the degree of insulin-mediated suppression of circulating non-esterified fatty acids (NEFAs) [17,45]. Briefly, %reduction of NEFA during the hyperinsulinemic euglycemic clamp study was calculated based on basal and nadir NEFA concentrations during the last hour of the glucose clamp study and adjusted by insulin concentration; this was used as an index of adipose tissue insulin sensitivity. Insulin clearance was calculated using the following equation [46,47]: insulin clearance = (IIR/[SS_SI_ − (B_SI_ ∗ SS_SC_/B_SC_)]), where IIR = insulin infusion rate, SS_SI_ = steady-state serum insulin concentration during the glucose clamp study, B_SI_ = basal serum insulin, SS_SC_ = steady-state serum C-peptide concentration during the glucose clamp study, and B_SC_ = basal serum C-peptide concentration.

### 2.4. H-Magnetic Resonance Spectroscopy

IMCL values of the right tibialis anterior (TA) and soleus (SOL) muscles and IHL values of liver segment 6 were measured using ^1^H-MRS (VISART EX V4.40, Toshiba, Tokyo) [48,49]. After the measurements, IMCL was quantified based on methylene signal intensity (S-fat), with creatine signal (Cre) as the reference. IMCL was calculated as the ratio of S-fat to Cre. IHL was quantified based on S-fat with H_2_O as the internal reference. IHL (%) was calculated as a percentage of H_2_O + S-fat [S-fat × 100/(H_2_O + S-fat)] [48,49].

### 2.5. Fat Distribution and Body Composition 

Intra-abdominal and subcutaneous fat areas were measured with MRI. Briefly, T1-weighted trans-axial scans were obtained and intra-abdominal and subcutaneous fat areas at the fourth and fifth lumbar interspaces were measured, as previously described using a specific software program (AZE Virtual Place, Tokyo, Japan) [49]. Body composition was assessed using the bioimpedance method (InBody; BIOSPACE, Tokyo, Japan).

### 2.6. Statistical Analysis

We used IBM SPSS Statistics for Windows, version 25.0. (IBM Corp., Armonk, NY, USA) for statistical analysis. Data are presented as means ± SD. Data were compared using the paired *t*-test for parametric data and the Wilcoxon signed-rank test for non-parametric data. All statistical tests were two-sided, with a significance level of 5%.

## 3. Results

### 3.1. Acute Effect Study

Table 1 summarizes the clinical features of the 12 participants. Mean age was 56.3 ± 7.6 years, mean duration of type 2 diabetes was 3.3 ± 1.5 years, and mean HbA1c was 7.7 ± 0.9%. Table 2 shows hyperinsulinemic euglycemic clamp data for the baseline study and acute effect study. EGP at 0 min was comparable between baseline study and acute effect study (Table 2). On the other hand, there were significantly larger changes in glucose, C-peptide, and insulin levels and significantly smaller changes in glucagon levels, NEFA levels, EGP, and urinary glucose excretion during the acute effect study compared with the baseline study (Supplementary Table S1) [38].

**Figure 2:**
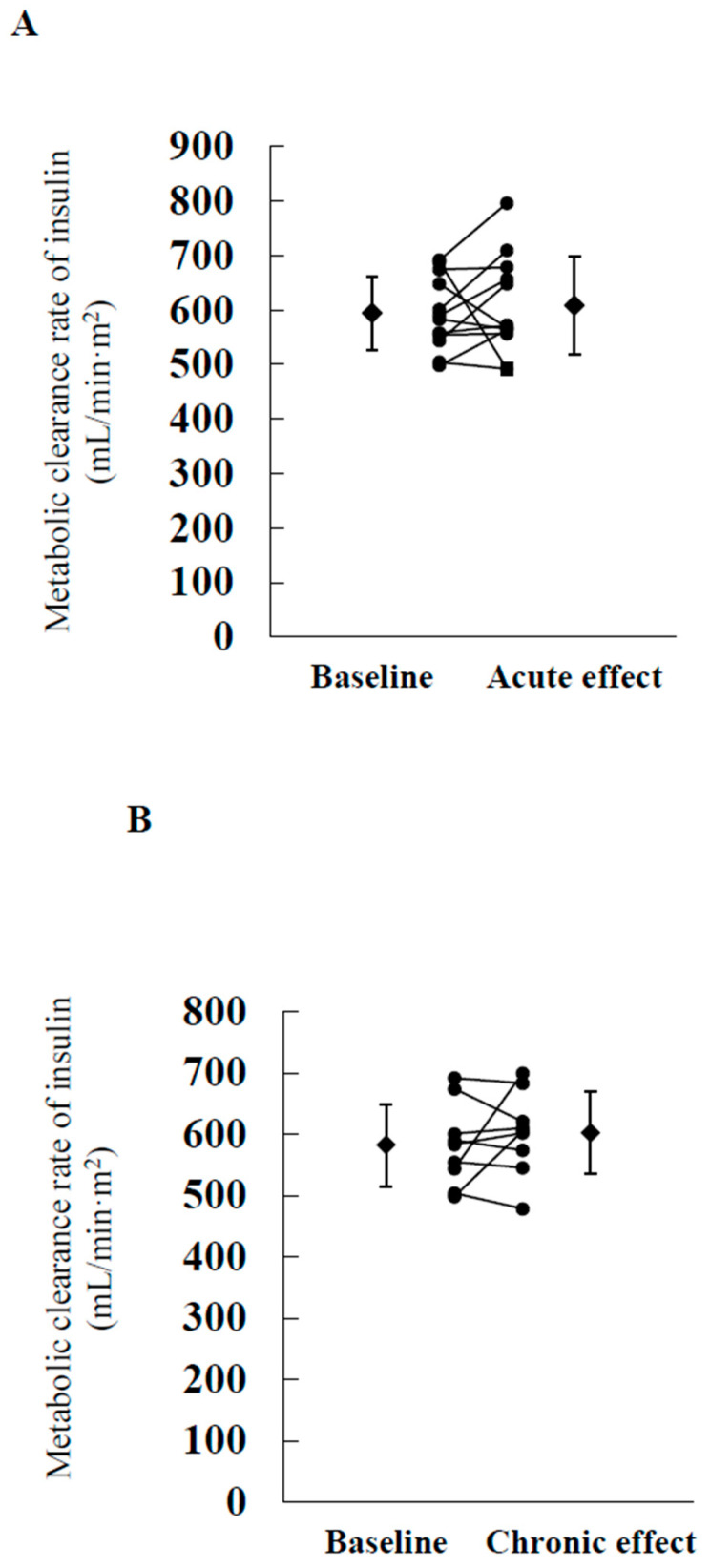
Changes in metabolic clearance rate of insulin in the acute effect study (**A**) and chronic effect study (**B**). (**A**) Metabolic clearance rate of insulin at baseline and after a single dose of tofogliflozin in 12 patients. (**B**) Metabolic clearance rate of insulin at baseline and after 8 weeks of treatment with tofogliflozin in nine patients.

MCRI levels measured during the hyperinsulinemic euglycemic clamp study were comparable between the baseline study and acute effect study (Table 2, Figure 2A). Rd was significantly higher, while muscle insulin sensitivity (TGU/SS_SI_) tended to be higher in the acute effect study compared with the baseline study. EGP at 360 min was significantly higher in the acute effect study compared with the baseline study. Percent reduction of EGP was significantly higher in the baseline study. However, hepatic insulin sensitivity (%reduction of EGP/SS_SI_) was similar. Adipose insulin sensitivity (%reduction of NEFA/SS_SI_) was comparable between baseline and acute effect study.

### 3.2. Chronic Effect Study

Nine participants completed this study protocol without any evident untoward effects. Table 3 shows the clinical features of the nine participants during the baseline study and chronic effect study. Tofogliflozin treatment significantly increased urinary glucose excretion and reduced HbA1c. During the fasting state, glucose levels were significantly decreased; however, insulin, C-peptide, NEFA, and glucagon levels did not change. In addition, tofogliflozin treatment significantly decreased body weight, fat mass, and abdominal subcutaneous fat area. Systolic and diastolic blood pressures were significantly decreased. On the other hand, IMCL in TA and SOL and IHL levels did not change significantly with treatment.

As shown in Figure 2B and Table 3, MCRI was similar after 8 weeks of tofogliflozin treatment. In addition, 8 weeks of tofogliflozin treatment significantly increased Rd. TGU and muscle insulin sensitivity (TGU/SS_SI_) tended to be higher after treatment, while hepatic insulin sensitivity (%reduction of EGP/SS_SI_) and adipose tissue insulin sensitivity (%reduction of NEFA/SS_SI_) did not change.

## 4. Discussion

Previous reports have demonstrated that a surrogate marker of insulin clearance during a meal load test is higher after a single dose of an SGLT2i or chronic treatment [34,35]. However, it remains unclear whether SGLT2i treatment alters systemic insulin clearance as measured by a hyperinsulinemic euglycemic clamp study. Thus, we investigated the effects of a single dose of tofogliflozin and 8 weeks of tofogliflozin treatment on MCRI as evaluated by a hyperinsulinemic euglycemic clamp study. MCRI did not change after a single dose of tofogliflozin (acute effect study). After an 8-week treatment with tofogliflozin, MCRI did not change, while body weight, fat mass, abdominal subcutaneous fat area, and HbA1c were significantly decreased and muscle insulin sensitivity tended to be increased (chronic effect study).

Previous studies showed that SGLT2i treatment increased insulin clearance during a meal load test [34,35]. The present study demonstrated that MCRI based on a hyperinsulinemic euglycemic clamp study was not altered after a single dose or 8 weeks of tofogliflozin. A previous study suggested that insulin clearance during an oral glucose tolerance test was correlated with MCRI measured with a glucose clamp study; however, the correlation coefficient was moderate (*r* = 0.74, *p* < 0.001) [50]. Thus, insulin clearance during a meal test is different from insulin clearance during a hyperinsulinemic euglycemic clamp study. Of note, it has been shown that insulin clearance in the postprandial state is reduced in parallel with the amount of insulin secreted [51], probably due to saturation of receptor-mediated endocytosis [51]. In fact, sulfonylureas, which strongly increase insulin release from beta-cells, reduce insulin clearance during a meal load test [52]. Therefore, it is possible that insulin clearance as evaluated by the insulin–C-peptide ratio in the postprandial state increases when endogenous insulin secretion decreases. Since lower postprandial insulin secretion after a single dose or chronic administration of an SGLT2i is probably due to decreased blood glucose levels [34,35], enhanced insulin clearance during a meal load test after SGLT2i administration might be due to decreased endogenous insulin secretion, not increased systemic insulin clearance.

Several studies have reported that SGLT2i treatment improved fatty liver [20,21,22,23,25,26]. For example, SGLT2i treatment lasting 12 to 72 weeks in patients with NAFLD and type 2 diabetes significantly reduces IHL accumulation, as evaluated by MRI-derived proton density fat fraction or ^1^H-MRS [20,21,22,23,25,26]. Compared with these studies, our study period was relatively short (8 weeks vs. 12–72 weeks), and the reduction in body weight was smaller (1.7% vs. 1.9–4.3%) [20,21,22,23,25], which might have contributed to the lack of change in IHL levels in the present study. In addition, urinary glucose excretion theoretically reduces IHL through suppressed de novo hepatic lipogenesis [53]. However, we observed elevation of NEFAs’ levels, which is known as a main source of IHL [54], after a single dose of tofogliflozin. A previous report also suggested that postprandial NEFA elevations at the beginning of SGLT2i treatment were probably due to decreased insulin levels [34]. Fasting NEFA levels were also elevated at 24 weeks after SGLT2i treatment in a large cohort [55]. Thus, one possibility is that NEFA elevation during SGLT2i treatment might partly inhibit the effects of SGLT2i on IHL reduction. In addition, fatty liver is associated with decreased insulin clearance [56,57]; thus, unaltered IHL levels might have also contributed to unchanged MCRI in the present study.

This study had a relatively short treatment period. However, previous studies showed that SGLT2i treatment increased insulin clearance immediately during a meal load test [34]. In addition, only a 3-day, low-carbohydrate diet increased MCRI, as measured by a hyperinsulinemic euglycemic clamp [37]. Thus, even very short-term treatment with SGLT2i is expected to increase MCRI, because, similar to the effects of a low-carbohydrate diet, SGLT2i promotes urinary excretion of glucose. Furthermore, animal experiments suggest that central obesity and fatty liver induced by a high-fat diet are prevented when a decrease in insulin clearance is inhibited [7,8]. Thus, we hypothesized that MCRI increases very early after initiation of SGLT2i treatment and leads to improvements in other metabolic parameters; this was not supported by the present study. Thus, the present study suggests that decreased levels of body fat and improved glycemic control with SGLT2i treatment occur independently of changes in MCRI.

There are several limitations in this study. First, we recruited only Japanese men. Metabolic clearance of insulin has been shown to differ by ethnicity and sex [58,59]. Therefore, it remains unclear whether our data can be applicable in persons of other ethnicities or women. Second, the number of participants in this study was relatively small. However, we observed significant decreases in body weight, body fat, and HbA1c, simultaneously, after an 8-week tofogliflozin treatment, while the MCRI level was unchanged. Thus, the present study could suggest that an increase in MCRI does not precede decreased body fat or improved glycemic control during SGLT2i treatment. Finally, we did not measure tissue-specific insulin clearance. SGLT2i mainly inhibits SGLT2 in kidney; thus, insulin clearance in kidney may be particularly relevant. A very recent report has shown that a high-fat diet increases renal insulin clearance to limit hyperinsulinemia in rats, while a high-fat diet modestly decreases hepatic insulin clearance [60], suggesting opposite tissue-specific insulin clearance in kidney and liver could occur after body weight changes and systemic insulin clearance is determined. However, no studies have investigated the effect of SGLT2i on tissue-specific insulin clearance. Further study is clearly required to clarify the effect.

In conclusion, MCRI as measured with a hyperinsulinemic euglycemic clamp study did not change after a single dose or 8 weeks of administration of tofogliflozin. Thus, the present study suggests that an increase in MCRI does not precede decreased body fat or improved glycemic control during SGLT2i treatment.

## Figures and Tables

**Table 1:** Clinical characteristics of study participants at baseline (*n* = 12).

Age (years)	56.3 ± 7.3
Duration of diabetes (years)	3.3 ± 1.4
Weight (kg)	72.6 ± 6.9
BMI (kg/m^2^)	25.0 ± 2.4
% body fat	26.6 ± 5.2
Systolic blood pressure (mmHg)	142.6 ± 18.1
Diastolic blood pressure (mmHg)	86.7 ± 11.2
Fasting glucose (mmol/L)	8.88 ± 1.37
Fasting insulin (pmol/mL)	83.9 ± 45.9
Glycated hemoglobin (%)	7.7 ± 0.8

Data are expressed as means ± SD.

**Table 2:** Hyperinsulinemic euglycemic clamp data in the baseline and acute effect studies.

.	Baseline Study	Acute Effect Study	*p*
SS_SI_ (average from 340–360 min) (pmol/L)	503.7 ± 53.1	499.4 ± 76.8	0.811
EGP (0 min) (mg/kg·min^−1^)	1.95 ± 0.18	1.99 ± 0.19	0.435
EGP (180 min) (mg/kg·min^−1^)	1.73 ± 0.14	2.07 ± 0.25	<0.001
EGP (360 min) (mg/kg·min^−1^)	0.21 ± 0.20	0.36 ± 0.13	0.022
% reduction of EGP (%)	89.3 ± 10.7	81.2 ± 7.0	0.032
% reduction of EGP/SS_SI_ (%/μU·mL⁻^1^)	1.29 ± 0.24	1.19 ± 0.19	0.247
Rd (mg/kg FFM·min^−1^)	4.11 ± 2.44	5.35 ± 2.10	0.008
TGU (mg/kg FFM·min^−1^)	4.11 ± 2.44	4.50 ± 2.12	0.209
TGU/SS_SI_ (μg/kg FFM·min^−1^ μU^−1^·mL)	59.0 ± 33.2	67.4 ± 36.5	0.080
% reduction of NEFA (%)	78.4 ± 13.2	83.9 ± 6.6	0.093
%reduction of NEFA/SS_SI_ (%/μU·mL⁻^1^)	1.13 ± 0.23	1.23 ± 0.22	0.158
MCRI (mL/min per m^2^)	594.7 ± 19.6	608.3 ± 26.2	0.605

Data are expressed as means ± SD. SS_SI_, steady-state serum insulin; EGP, endogenous glucose production; Rd, rate of the disappearance of glucose; TGU, rate of tissue glucose uptake; FFM, fat-free mass; NEFA, non-esterified fatty acid; MCRI, metabolic clearance rate of insulin during a hyperinsulinemic euglycemic clamp study.

**Table 3:** Various clinical parameters at baseline and after 8 weeks of tofogliflozin treatment (chronic effect study) (*n* = 9).

	Baseline Study	Chronic Effect Study	*p*
Weight (kg)	73.2 ± 7.3	72.0 ± 7.0	0.013
BMI (kg/m^2^)	25.3 ± 2.6	24.9 ± 2.5	0.012
% body fat	26.7 ± 5.9	24.9 ± 4.7	0.073
Free fat mass (kg)	53.5 ± 4.8	53.9 ± 4.5	0.359
Fat mass (kg)	19.8 ± 5.4	18.1 ± 4.5	0.046
Systolic blood pressure (mmHg)	146.0 ± 18.0	125.1 ± 11.1	<0.001
Diastolic blood pressure (mmHg)	87.6 ± 11.7	77.6 ± 10.8	0.003
glycated hemoglobin (%)	7.7 ± 0.9	7.0 ± 0.8	0.001
Fasting glucose (mmol/L)	9.2 ± 1.9	7.9 ± 0.9	0.025
Fasting insulin (pmol/L)	78.9 ± 43.1	64.6 ± 40.9	0.110
Fasting C-peptide (ng/L)	2.5 ± 0.8	2.3 ± 0.9	0.395
Fasting glucagon (ng/L)	51.5 ± 15.4	45.0 ± 17.5	0.205
Fasting free fatty acids (μmol/L)	979.1 ± 239.3	1001.2 ± 296.2	0.816
Adiponectin (μg/mL)	6.6 ± 2.7	6.2 ± 1.9	0.381
Triglyceride (mmol/L)	2.5 ± 1.3	3.1 ± 3.2	0.859
High-density lipoprotein cholesterol (mmol/L)	1.4 ± 0.7	1.4 ± 0.5	0.678
Low-density lipoprotein cholesterol (mmol/L)	3.3 ± 0.8	3.2 ± 1.3	0.668
Aspartate transaminase (IU/L)	26.7 ± 8.4	25.4 ± 10.1	0.310
Alanine aminotransferase (IU/L)	37.7 ± 16.5	29.4 ± 9.4	0.056
High-sensitivity CRP (mg/dL)	702.4 ± 318.9	663.4 ± 523.8	0.678
Acetoacetic acid (µmol/L)	56.1 ± 26.0	87.9 ± 71.2	0.374
1.3-hydroxybutyric acid (µmol/L)	96.9 ± 67.1	178.6 ± 170.1	0.314
Total ketone body (µmol/L)	153.0 ± 92.7	266.4 ± 240.7	0.314
Urinary glucose excretion (mmol/3 h)	3.6 ± 7.5	214.6 ± 160.6	0.004
Abdominal visceral fat area (cm^2^)	191.2 ± 38.9	172.2 ± 32.9	0.060
Abdominal subcutaneous fat area (cm^2^)	194.7 ± 66.2	182.3 ± 65.9	0.043
Intrahepatic lipid (%)	17.6 ± 8.1	18.0 ± 8.4	0.893
Intramyocellular lipid in TA (S-fat/Cre)	4.9 ± 2.9	4.9 ± 2.5	0.933
Intramyocellular lipid in SOL (S-fat/Cre)	11.1 ± 4.6	8.3 ± 2.3	0.089
EGP (0 min) (mg/kg·min^−1^)	1.94 ± 0.19	2.23 ± 0.35	0.002
EGP (360 min) (mg/kg·min^−1^)	0.26 ± 0.14	0.37 ± 0.32	0.373
% reduction of EGP (%)	86.1 ± 7.7	87.8 ± 3.4	0.564
% reduction of EGP/SS_SI_ (%/μU·mL⁻^1^)	1.22 ± 0.18	1.29 ± 0.19	0.307
Rd (mg/kg FFM·min^−1^)	4.26 ± 2.63	5.35 ± 2.73	0.035
TGU (mg/kg FFM·min^−1^)	4.25 ± 2.63	5.15 ± 2.74	0.059
TGU/SS_SI_ (μg/kg FFM·min^−1^ μU^−1^·mL)	60.5 ± 36.0	77.4 ± 50.8	0.054
% reduction of NEFA (%)	76.3 ± 14.6	82.8 ± 9.7	0.116
% reduction of NEFA/SS_SI_ (%/μU·mL⁻^1^)	1.09 ± 0.25	1.21 ± 0.23	0.260
MCRI (mL/min per m^2^)	582.5 ± 22.4	602.3 ± 22.3	0.405

Data are expressed as means ± SD. CRP, C-reactive protein; TA, tibialis anterior; SOL, soleus; S-fat, methylene signal intensity; Cre, creatine; EGP, endogenous glucose production; SS_SI_, steady-state serum insulin; Rd, rate of the disappearance of glucose; TGU, rate of tissue glucose uptake; FFM, fat-free mass; NEFA, non-esterified fatty acid; MCRI, metabolic clearance rate of insulin.

## Data Availability

The data presented in this study are available on request from the corresponding author. The data are not publicly available due to privacy.

## Supplementary Materials

The following are available online at https://www.mdpi.com/article/10.3390/biomedicines9091154/s1, Table S1: Changes in each parameter from 0 to 180 min.
