# Supplementary material for: Short-Term SGLT2 Inhibitor Administration Does Not Alter Systemic Insulin Clearance in Type 2 Diabetes
Source: Biomedicines. 2021 Sep 3;9(9):1154. doi: 10.3390/biomedicines9091154 (PMC8472728; doi:10.3390/biomedicines9091154)
Supplement: Supplementary file 1 [file biomedicines-09-01154-s001.zip › biomedicines-1326436-supplementary.pdf]

Supplementary Table S1. Changes in each parameter from 0 to 180 minutes

	Baseline study	Acute effect study	*P*
Glucose (mg/dL)	-14.6 ± 13.3	-26.4 ± 14.6	**.002**
Insulin (μU/mL)	-0.4 ± 1.8	-1.8 ± 1.6	**.019**
C-peptide (ng/mL)	-0.1 ± 0.2	-0.3 ± 0.2	**.001**
Glucagon (pg/mL)	5.1 ± 4.5	11.1 ± 6.6	**.015**
NEFA (μEq/L)	86.0 ± 74.9	198.5 ± 149.5	**.017**
EGP (mg/kg·min^{-1})	-0.22 ± 0.12	0.08 ± 0.20	**<.001**
Urinary glucose excretion (mg/3 hours)	51.8 ± 112.3	7845.7 ± 2720.2	**<.001**

Data are expressed as means ± SD or medians (interquartile range).

NEFA, non-esterified fatty acids; EGP, endogenous glucose production.
